# Development of a metabolic pathway transfer and genomic integration system for the syngas-fermenting bacterium *Clostridium ljungdahlii*

**DOI:** 10.1186/s13068-019-1448-1

**Published:** 2019-05-08

**Authors:** Gabriele Philipps, Sebastian de Vries, Stefan Jennewein

**Affiliations:** 1 Department for Industrial Biotechnology, Fraunhofer IME, Fraunhofer Institute for Molecular Biology and Applied Ecology IME, Forckenbeckstr. 6, 52074 Aachen, Germany; 2 Present Address: Department of Intensive Care Medicine, University Hospital, RWTH Aachen University, Pauwelsstr. 30, 52074 Aachen, Germany

**Keywords:** Synthetic biology, Metabolic engineering, Acetogen, Conjugation, Inducible promoter, Himar1 transposase, Genomic integration, Syngas fermentation, Isopropanol, Acetone, Bio-fuels

## Abstract

**Background:**

*Clostridium* spp. can synthesize valuable chemicals and fuels by utilizing diverse waste-stream substrates, including starchy biomass, lignocellulose, and industrial waste gases. However, metabolic engineering in *Clostridium* spp. is challenging due to the low efficiency of gene transfer and genomic integration of entire biosynthetic pathways.

**Results:**

We have developed a reliable gene transfer and genomic integration system for the syngas-fermenting bacterium *Clostridium ljungdahlii* based on the conjugal transfer of donor plasmids containing large transgene cassettes (> 5 kb) followed by the inducible activation of *Himar1* transposase to promote integration. We established a conjugation protocol for the efficient generation of transconjugants using the Gram-positive origins of replication *repL* and *repH*. We also investigated the impact of DNA methylation on conjugation efficiency by testing donor constructs with all possible combinations of Dam and Dcm methylation patterns, and used bisulfite conversion and PacBio sequencing to determine the DNA methylation profile of the *C. ljungdahlii* genome, resulting in the detection of four sequence motifs with N^6^-methyladenosine. As proof of concept, we demonstrated the transfer and genomic integration of a heterologous acetone biosynthesis pathway using a *Himar1* transposase system regulated by a xylose-inducible promoter. The functionality of the integrated pathway was confirmed by detecting enzyme proteotypic peptides and the formation of acetone and isopropanol by *C. ljungdahlii* cultures utilizing syngas as a carbon and energy source.

**Conclusions:**

The developed multi-gene delivery system offers a versatile tool to integrate and stably express large biosynthetic pathways in the industrial promising syngas-fermenting microorganism *C. ljungdahlii*. The simple transfer and stable integration of large gene clusters (like entire biosynthetic pathways) is expanding the range of possible fermentation products of heterologously expressing recombinant strains. We also believe that the developed gene delivery system can be adapted to other clostridial strains as well.

**Electronic supplementary material:**

The online version of this article (10.1186/s13068-019-1448-1) contains supplementary material, which is available to authorized users.

## Introduction

The microbial conversion of industrial waste streams into bulk chemicals and fuels is a promising approach to reduce greenhouse gas emissions and offset the effects of climate change [1]. The bacterial genus *Clostridium* includes several species that can produce valuable chemical building blocks, such as acetone, organic acids, and various short/medium-chain alcohols [2]. For industrial-scale production, the feedstock is often starchy plant material that provides sugars as a carbon and energy source, e.g., *C. acetobutylicum* can produce acetone using starch as a substrate [3]. However, one drawback is that starch crops cultivated for industrial purposes compete with food and feed crops. A more attractive feedstock is synthesis gas (syngas), a mixture of carbon monoxide, carbon dioxide, and hydrogen, which is emitted in large amounts e.g., by the steel industry and can be produced by the gasification of municipal waste [4]. *C. ljungdahlii* and *C. autoethanogenum* among others can utilize syngas as a sole carbon and energy source [4, 5]. These species use the reductive acetyl-CoA pathway, also known as the Wood–Ljungdahl pathway, to reduce CO, CO_2_, and H_2_ to the key metabolite acetyl-CoA, which is processed further into biomass and the main fermentation products ethanol and acetate [6–8].

The ability of certain *Clostridium* species to convert syngas into acetyl-CoA, a central biochemical building block, could be exploited to produce additional chemicals by extending and modifying the corresponding metabolic pathways. However, the genetic manipulation of Gram-positive bacteria remains much more challenging than the amenable processes developed for model Gram-negative species such as *Escherichia coli*, due to the physiological and biochemical distinctions between these groups of bacteria. Successful metabolic engineering in Gram-positive bacteria requires a strategy to achieve the introduction of multiple transgenes encoding appropriate enzymes and to ensure their stable integration and expression, while overcoming the restriction–methylation system of the host.

Multi-gene transformation in Gram-positive bacteria can be achieved using traditional approaches such as electroporation, but the efficiency of gene transfer tends to be inversely proportional to the plasmid size, as shown in other species such as *E. coli* [9, 10]. Optimal parameters must be established empirically or systematically for each species [11]. An alternative strategy is conjugation, a natural DNA transfer process that requires only the presence of a dedicated origin of transfer (*oriT*) on the donor plasmid, with all other functions (*tra* and *mob* genes) provided in *trans* [12]. Conjugation is versatile because it mobilizes large plasmids efficiently and can transfer DNA between species, including transfer between Gram-negative and Gram-positive bacteria [13] and from bacteria to eukaryotes [14]. This method has been used for DNA transfer to *C. beijerinckii* NCIMB 8052 (formerly *Clostridium acetobutylicum* NCIB 8052) [15] and *C. perfringens* [16] and is the standard method for DNA transfer to *C. difficile* [17]. Although conjugation is an efficient strategy for gene transfer to *Clostridium* spp., the donor plasmid is susceptible to the host’s restriction–modification system [18]. The decline in conjugation efficiency caused by unmethylated recognition sites within the donor plasmid sequence has been demonstrated for *C.* *difficile* [17]. Plasmid instability is also an important issue, especially in an industrial context where the bulk use of antibiotics to maintain selection pressure is too expensive [19, 20]. These issues can be addressed by targeted genomic integration, as has been demonstrated using the ClosTron system based on group II introns [21]. Various transposon systems have also been used to achieve non-targeted integration, including the conjugative transposon *Tn*916 [22–24] and non-conjugative transposons such as *Tn*5 [25, 26]. While most approaches use transposon mutagenesis as a means for reverse genetics to disrupt genes [23–25, 27], it has also been applied to integrate entire biosynthetic pathways into several Gram-negative bacteria species [26, 28]. A useful alternative is the mariner-type transposon *Himar1* from *Haematobia irritans* [29], which has the capability to deliver biosynthetic pathways as large as 29.6 and 57.5 kb into the genome [30]. The *Himar1* transposase facilitates the random and even multiple integration of DNA sequences flanked by ~ 27-bp inverted terminal repeats (ITRs) without additional cofactors [29], making the system applicable in any host including *Clostridium* spp. [31]. The integration occurs by a ‘cut and paste’ mechanism in the 2-bp TA target sequence of Himar1, which is duplicated upon insertion [32]. The *Staphylococcus xylosus* xylose-inducible promoter–repressor system [33] is functional in *C. acetobutylicum* [34] and can be used to induce the transposase. After conjugal transfer of the donor plasmid, the addition of xylose induces *Himar1* transposase expression and promotes the integration of one or more copies of the cassette bounded by the ITRs for increased transgene stability, with multiple integration events enhancing the gene dosage and ultimately the anticipated product yield.

Here we describe the development of an efficient conjugation and genomic integration protocol that allows the transfer of large gene cassettes, e.g. coding for entire metabolic pathways, to the host species *C. ljungdahlii* and thus facilitates the production of different bulk chemicals and fuels from a syngas-fermenting bacterial strain. The industrial scaling of such a process would allow the recycling of syngas, reducing the environmental harm caused by its release, and channeling this waste stream into the production of valuable chemicals.

## Results and discussion

### Development and optimization of a conjugation protocol for *C. ljungdahlii*

The syngas-fermenting bacterium *C. ljungdahlii* offers the tantalizing prospect of converting an important industrial waste stream into environmentally sustainable platform chemicals and fuels. Although the availability of a complete *C. ljungdahlii* genome sequence will facilitate this process [35], several technical challenges must be addressed before efficient metabolic engineering is possible, including the low efficiency of transformation with large gene cassettes [36]. The latter reflects a combination of factors including inefficient gene transfer, a hostile host restriction–modification system, and the instability of plasmid DNA.

We addressed these challenges by developing a robust gene delivery system based on conjugation, which efficiently transfers large plasmids, combined with an inducible *Himar1* transposase to facilitate cassette integration into the *C. ljungdahlii* genome. To establish and optimize the conjugation procedure for *C. ljungdahlii*, the *oriT* region of the broad host range IncPα plasmid RK2 [37, 38] was transferred to a derivative of pSOS95 vector, which contains the *repL* (pIM13) origin of replication for Gram-positive bacteria [34, 39], resulting in the vector pSOS-traJ. Several further origins of replication are suitable for *C. ljungdahlii* [40], and to extend the potential of our conjugation platform we therefore replaced *repL* with the broad-range origin *repH* (pCB102) to generate the final vector pSOS-traJ-repH.

**Fig. 1:**
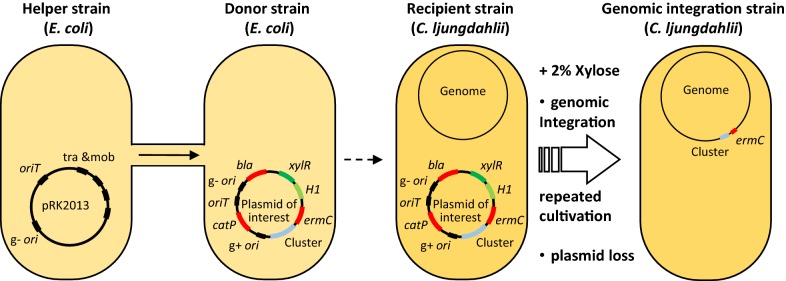
Schematic representation of the triparental mating and genomic integration process. A helper strain (*E. coli* Stbl3) is used to provide the genes necessary for conjugation in *trans* on the helper plasmid pRK2013. Once the helper plasmid is transferred through the conjugation bridge, the donor strain with the plasmid of interest can transfer its cargo to the recipient *Clostridium* strain via conjugation. The mechanism of plasmid transfer between Gram-negative and Gram-positive bacteria is currently unknown and is indicated by a dotted arrow. Genomic integration is induced by the addition of 2% xylose to the medium. Plasmid loss is promoted by repeated cultivation in YTF medium with selection on d-cycloserine and clarithromycin only, omitting thiamphenicol

Triparental mating was carried out using donor and helper strains of *E. coli* and the recipient strain of *C. ljungdahlii* (Fig. 1). We used the *E. coli* B-strain NEB Express as a donor, since it was reported that B-strains achieve higher *C. ljungdahlii* transformation efficiencies by electroporation than K-strains [40]. The helper strain Stbl3 carries the vector pRK2013, which supplies the necessary *tra* and *mob* functions in *trans* [41].

Several conjugation methods were tested with variable results. The most straight-forward method (concentrating, washing, and incubating the mixed strains in liquid medium overnight, as often used with *E. coli*) was unsuccessful. Switching to solid rich medium achieved better results as suggested by Willets [42], although the frequency of transconjugant *C. ljungdahlii* colonies varied widely among experimental replicates. We therefore investigated the relationship between the conjugation efficiency and culture volume by conducting parallel triparental mating experiments in liquid medium and on solid medium poured either 2 days or 2 weeks before conjugation, which results in the absorption of different quantities of the concentrated conjugation broth. We also varied the amount of conjugation broth applied to the plates (100 µl or 1 ml).

**Fig. 2:**
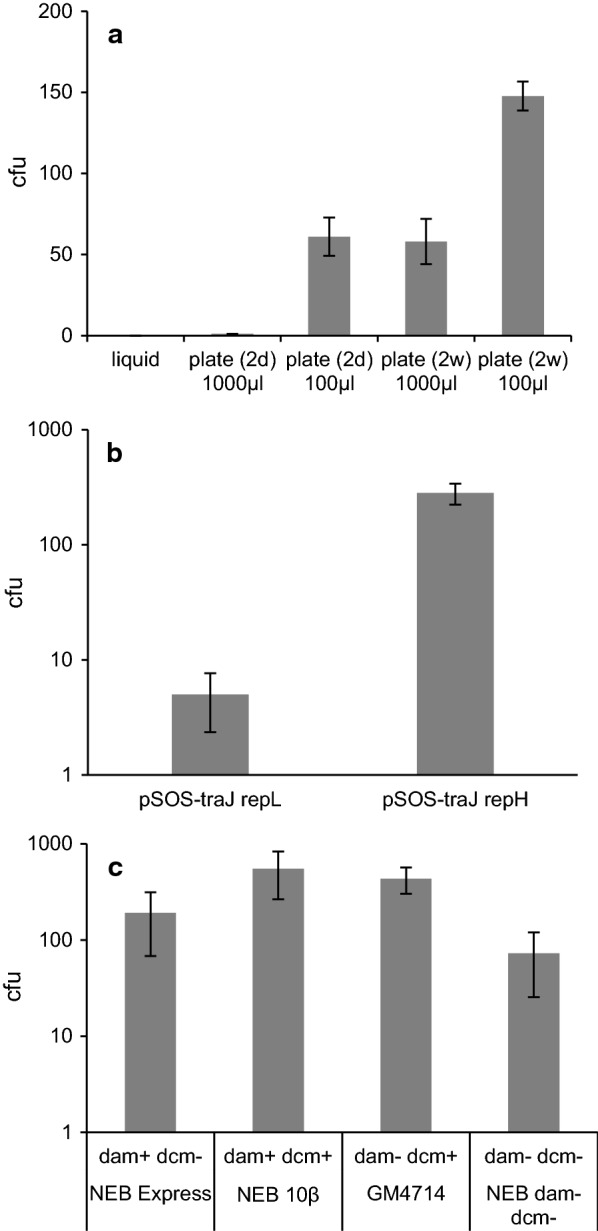
Number of transconjugants per plate when using different mating setups. **a** The same number of cells was subjected to different mating conditions. Conjugation was performed in liquid medium, applied as 1000 µl or 100 µl onto freshly poured plates (2 days) or as 1000 µl or 100 µl onto 2-week-old well-dried YTF agar plates (2 weeks). **b** Conjugation was performed using donor plasmids with the *Clostridium* origin of replication *repL* and *repH*. **c** *E. coli* strains with different *dam* and *dcm* methylation patterns were used as the donor strain containing pSOS-traJ repH. The data represent biological triplicates with standard deviations

The lower mating culture volume (100 µl) on a fresh plate gave comparable results to plating the higher volume (1 ml) onto older plates, but the combination of the lower mating culture volume and the well-dried plate for overnight mating gave the best results (Fig. 2a). Placing unsealed fresh plates face down at 37 °C for 1–2 days accelerated the drying process and also increased the number of transconjugants (unpublished observations). The growth of freshly transformed *C. ljungdahlii* can be poor on the plate or is inhibited by selection pressure, so the harvested cultures were poured into the plates after mating, using low-melting-point agarose to prevent heat shock. The impact of these minor procedural differences may explain the range of conjugation frequencies reported by different research groups using an identical protocol [43].

To verify the complete transfer of the donor plasmid, 10 transconjugant colonies were picked randomly and the isolated plasmid DNA was reintroduced into *E. coli* for restriction analysis. Digestion of the plasmids with *Hin*dIII revealed the anticipated restriction pattern (unpublished observations).

### The impact of different origins of replication

We compared two different origins of replication that are known to function in *C. ljungdahlii* and other Gram-positive bacteria: *repL* (replicon pIM13, derived from *Bacillus subtilis*) and *repH* (replicon pCB102, derived from *C. butyricum*). Both origins were used with the same donor plasmid backbone, pSOS-traJ. The *repH* origin was the most efficient, generating two orders of magnitude more transconjugant *C. ljungdahlii* colonies than *repL* (Fig. 2b). This supports the results achieved using the same origins with an electroporation protocol [40] although no firm conclusions can be drawn because the vector backbones and selection procedures were different in this earlier study. It is unclear whether the better performance of the *repH* origin reflected its impact on mating efficiency (i.e., transfer) or stability (i.e., replication and maintenance). Our experiments indicate the latter is more likely, given the larger number of *repH* transformants recovered by plasmid rescue and the tendency for *repL* transformants to undergo a sudden arrest in cell growth after several passages. The instability of *repL* plasmids in *C. ljungdahlii* has been reported before [40, 44]. However, the instability of *repL* plasmids can be of advantage, when limited plasmid stability is desirable.

A typical mating efficiency in our hands was 2.4 × 10^−6^ transconjugants per recipient cell. This is in the range reported for inter-species transfer especially between Gram-positive and Gram-negative bacteria. For example, Strätz et al. [45] reported a transfer efficiency of ~ 7.3 × 10^−6^ per recipient when transferring a plasmid from *E. coli* to *Acetobacterium woodii*. Efficiencies ranging from 4.7 × 10^−5^ to less than 3.3 × 10^−9^ per recipient have been reported for conjugation between *E. coli* and *C. beijerinckii* NCIMB 8052 (formerly *C. acetobutylicum* NCIB 8052) [15]. Although successful conjugation has been reported between *E. coli* and *C. cellulolyticum* [46], *C. difficile* [17], and *C. perfringens* [16], conjugation has not yet been achieved between *E. coli* and *C. acetobutylicum* ATCC 824 [47].

### The influence of donor strain dam and dcm methylation on conjugation efficiency

Host DNA is often protected from endogenous restriction endonucleases by modification, which typically involves the methylation of certain bases at the enzyme’s recognition site [18]. The efficiency of *C. ljungdahlii* transformation by electroporation was reported to be higher when using plasmids isolated from an *E.* *coli* B-strain with Dam methylase activity but lacking Dcm methylase activity (*dam*^+^*dcm*^−^) compared to the same plasmids isolated from a K strain (*dam*^−^*dcm*^+^) [40]. Furthermore, both Dam and Dcm methylation inhibit transformation and conjugation in *C. pasteurianum* NRRL B-598 [48], and Dcm methylation reduces the efficiency of transformation by electroporation in *C. thermocellum* DSM1313 [49]. Dam methylase modifies adenosine residues in the GATC motif, whereas Dcm methylase modifies the second cytidine residue in the CCWGG motif. To investigate the effect of *dam* and *dcm* methylation backgrounds on conjugation and colony forming efficiency, *E. coli* strains with all four possible combinations of *dam* and *dcm* methylation were transformed with pSOS-traJ-repH and used as the donor strain for conjugation (Fig. 2c). Although the *dam*^+^*dcm*^+^ strain generated the most colonies and the *dam*^−^*dcm*^−^ strain generated the fewest, the differences were not extensive and we conclude that (at least under our conditions) Dam and Dcm methylation do not substantially influence the success of conjugation between *E. coli* and *C. ljungdahlii.* Our data suggest that *C. ljungdahlii* has no adverse restriction methylation system or alternatively that conjugation circumvents the restriction system due to its single stranded DNA transfer mechanism and rapid methylation of the newly synthesized double stranded DNA [50]. Nevertheless, we recommend the cloning strain NEB10β (*dam*^+^*dcm*^+^) not only for its superior performance but also because it has an *endA1* and *recA* genotype, which abolishes nonspecific endonuclease I activity and homologous recombination.

### DNA methylation profiling using bisulfite conversion and PacBio sequencing

Another potential drawback of the diverse restriction–modification systems of different bacteria [51] is the unpredictable impact of further host DNA restriction enzymes on heterologous transgenes. We therefore investigated the *C. ljungdahlii* global DNA methylation profile to identify unique characteristics of this species that might affect conjugation efficiency or transgene expression. Bacterial genomes often contain 5-methylcytidine (m5C), N^4^-methylcytidine (m4C), and/or N^6^-methyladenosine (m6A), and various methods are available to map these residues [52]. Putative m5C residues were mapped by sequencing after bisulfite conversion, a deamination reaction that converts unmethylated and therefore unprotected cytidine residues to uridine and thus changes the base-paring properties at that site compared to the reference genome. However, comparison with the reference sequence for *C. ljungdahlii* DSM 13528 (GenBank CP001666.1) revealed no evidence for the presence of m5C residues. Putative m6A residues were mapped by PacBio sequencing, using a culture harvested in the logarithmic growth phase (Table 1). The most prominent motif was C^**m6**^**A**GNNNNNGAT (25% of potential sites methylated) together with its reverse complement ^**m6**^**A**TCNNNNNCTG (21% of potential sites methylated). Other sites were methylated at lower frequencies: GAAT^**m6**^**A**YC (18%), GATA^**m6**^**A**T (13%), and CAAAA^**m6**^**A**R (11%). The data were submitted to REBASE (http://rebase.neb.com) [53].

**Table 1:** Results of PacBio sequencing

Motif and modified position	Modification type	Number of modified motifs detected	Number of motifs in genome	Percentage of modified motifs detected (%)
C**A**GNNNNNGAT	m6A	581	2338	24.85
**A**TCNNNNNCTG	m6A	494	2338	21.13
GAAT**A**YC	m6A	251	1375	18.25
GATA**A**T	m6A	740	5769	12.83
CAAAA**A**R	m6A	504	4717	10.68

Summary of the detected motifs with methylated adenine residues (underlined)

The *C. ljungdahlii* methylation profile is therefore similar to that of *C. autoethanogenum*, which features m6A residues in the motifs CAAAA^**m6**^**A**R, GWTA^**m6**^**A**T, and SNNGCA^**m6**^**A**T [54] probably due to its close evolutionary relationship with *C. ljungdahlii* [55]. However, the above-mentioned motifs showed a methylation frequency of more than 85% in *C. autoethanogenum* [54]. The low frequency of methylated sites in our sample may reflect the testing of samples in the logarithmic growth phase, which may show limited methylation due to concurrent DNA replication [56]. Interestingly, the C^**m6**^**A**GNNNNNGAT and ^**m6**^**A**TCNNNNNCTG sites that are methylated in *C. ljungdahlii* are unmethylated in *C. autoethanogenum*. These motifs match the anticipated target sites for type I restriction–methylation systems, which comprise separate methylation, sequence recognition, and endonuclease domains [57], predicted to be represented by the proteins M.CljII (CLJU_c03310), S.CljII (CLJU_c03320), and CljIIP (CLJU_c03330) in *C. ljungdahlii*, respectively [53].

### Design of a versatile *E. coli*–*C. ljungdahlii* shuttle vector for genomic integration

**Fig. 3:**
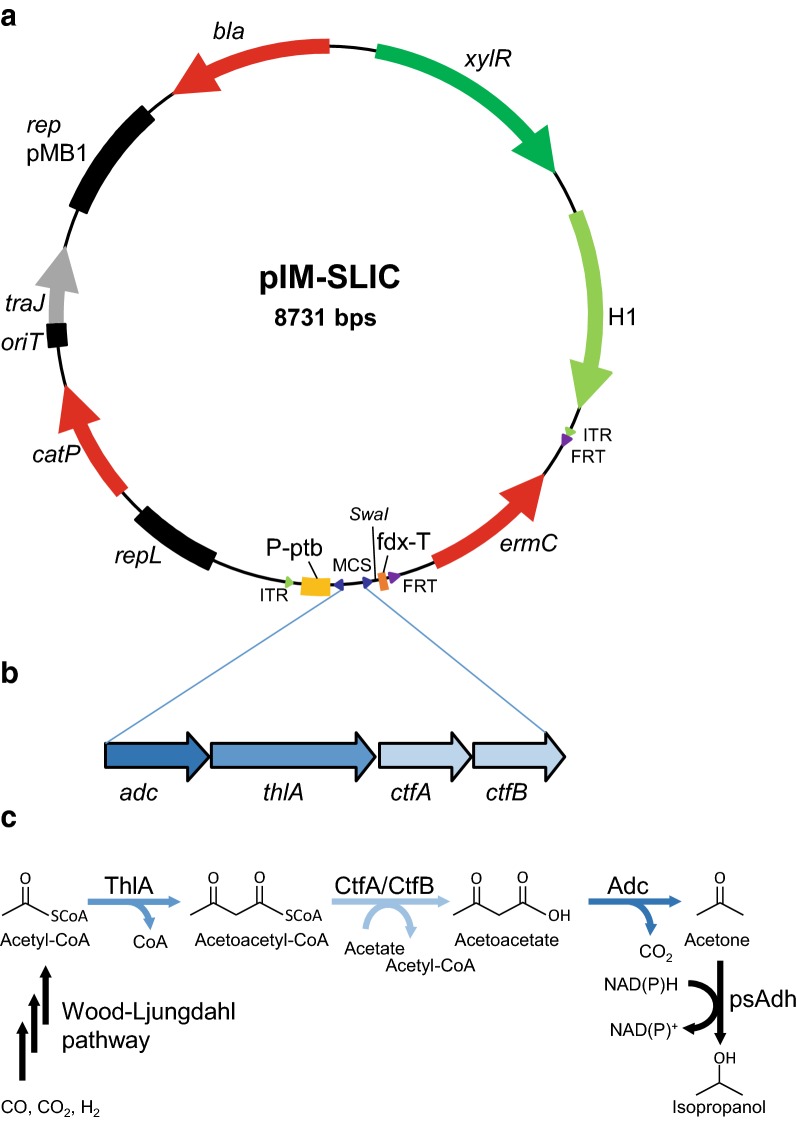
*E. coli*-*Clostridium* shuttle vector for the inducible genomic integration of an acetone biosynthesis gene cluster. **a** The vector pIM-SLIC contains a multiple cloning site (MCS) and a *Swa*I restriction site to allow directed and undirected insertion of cassettes. **b** A cluster comprising the four genes necessary for acetone biosynthesis (*adc*, *thlA*, *ctfA*-*ctfB*) was inserted by Gibson assembly via the SLIC sites (dark blue triangles) between the *ptb* promoter (P-ptb, yellow) and the *fdx* terminator (fdx-T, orange) resulting in pIM-Ace#22. **c** In *C. ljungdahlii* acetyl-CoA is derived from syngas (CO, CO_2_, H_2_) via the Wood–Ljungdahl pathway. Acetyl-CoA is used as a starting material for acetone biosynthesis by the engineered pathway. Acetone biosynthesis starts with the condensation of two acetyl-CoA molecules to acetoacetyl-CoA and the release of one molecule of CoA by acetyl-CoA acetyltransferase (ThlA). The CoA is then transferred to an acetate molecule by the CoA-transferase (CtfA/CtfB) to yield acetoacetate. Finally, acetone is generated via a decarboxylation reaction catalyzed by acetoacetate decarboxylase (Adc). However, the obtained acetone gets reduced by an endogenous primary secondary alcohol dehydrogenase (psAdh) to the secondary alcohol isopropanol

DNA transfer by conjugation must be followed by the stable genomic integration of a transgene cassette in order to maintain the new phenotype in the absence of selection. We therefore developed a new *E. coli*–*C. ljungdahlii* shuttle vector for this purpose (Fig. 3a).

The shuttle vector includes a pMB1 origin of replication and *bla* selectable marker for high-copy-number maintenance and selection in *E. coli*, and a *repL* origin of replication for maintenance in *C. ljungdahlii.* Additionally to *bla*, the *catP* and *ermC* selectable markers are included because only they can be used for propagation in both *E. coli* and *C. ljungdahlii* (*catP* is located on the non-integrating vector backbone and *ermC* is present as part of the transgene cassette and is used for the further selection of integrants). The vector also contains a *Himar1* transposase gene controlled by a xylose-inducible promoter–repressor system [33], allowing the induction of transposase activity. The transgene cassette (including *ermC*) is flanked by ITRs, which act as the substrate for the transposase. The *ermC* gene is also flanked by flippase recognition target (*FRT*) sites recognized by FLP recombinase, to allow subsequent marker removal and additional rounds of integration with the same cassette or a different one. An *oriT*/*traJ* region allows plasmid transfer by conjugation. SLIC sites are also present to facilitate cloning strategies for the introduction of additional transgenes by Gibson assembly. A variant of the shuttle vector containing *repH* and a hyperactive *Himar1* transposase (H1C9) [58] was generated as well resulting in pIM-SLIC-H1C9-repH. As discussed above, the inclusion of a *repH* origin of replication instead of *repL* generated a larger number of transconjugants (Fig. 2b), which may rather reflect the ability of *repH* to maintain the plasmid as an episome. However, the use of the repL origin is more suitable when rapid vector loss is desirable.

### Proof of principle using a Clostridium acetone biosynthesis pathway

The new shuttle vector was tested by introducing a *clostridial* acetone biosynthesis pathway (Fig. 3b), comprising the genes *adc*, *thlA*, and *ctfA*-*ctfB*. The *C. acetobutylicum thl*A and *ctfA*-*ctfB* genes were used because this species is a natural producer of acetone [3]. However, the *C. acetobutylicum* Adc enzyme is inhibited by CO [59, 60], so we used the *C. beijerinckii adc* gene instead. The three corresponding enzymes catalyze the production of acetone from the intermediate acetyl-CoA (Fig. 3c). The gene cluster was placed under the control of the strong *C. acetobutylicum ptb* promoter and upstream of the *C. pasteurianum* DSM525 *fdx* transcriptional terminator. After verifying the nucleotide sequence of the vector by DNA sequencing, triparental mating was carried out as described above with *C. ljungdahlii* as the recipient strain. The 12-kb plasmid was successfully transferred to the recipient as shown by comparative restriction analysis after plasmid isolation and reintroduction into *E. coli* NEB10β (Additional file 1: Figure S1). Even larger constructs of 18, 20, and 25.6 kb were also successfully transferred by conjugation (unpublished observations).

For genomic integration, a single transconjugant containing pIM-Ace#22 was grown repeatedly in YTF medium containing clarithromycin and d-cycloserine for selection and 2% (w/v) xylose to induce transposase activity (Fig. 1). Subsequently, 100 µl of the suspension was spread onto YTF agar plates without xylose or antibiotics, and the resulting colonies were replica plated onto medium containing either clarithromycin or thiamphenicol to identify the integrants. Colonies containing episomal plasmids remain resistant to both antibiotics whereas integrants should be sensitive to thiamphenicol but resistant to clarithromycin. The latter colonies were picked and inoculated into fresh medium to allow the extraction and analysis of genomic DNA. The site of genomic integration was identified by inverse PCR (Additional file 2: Figure S2). Strain Ace#22-24 was investigated in more detail, revealing that the cassette had integrated within a gene encoding a putative membrane protein (CLJU_c32170) at position 3523326. An analytical PCR confirmed the integration at this position and loss of the plasmid backbone (Additional file 3: Figure S3). Whole-genome sequencing was carried out to verify the site of integration, revealing the integration of a single copy of the complete 5-kb cassette comprising the acetone cluster and the *ermC* selectable marker between two ITRs. Successful genomic integration was also confirmed for an 11.1-kb fragment (unpublished observations) indicating that *Himar1* transposase can facilitate the integration of even larger DNA fragments into the *C*. *ljungdahlii* genome.

Although *Himar1* transposase has mostly been used for the generation of random mutagenesis libraries [31, 61, 62] and also for complementation in a mutant *Rickettsia rickettsii* strain [63], we have shown for the first time that the same system can be used for the introduction of several genes corresponding to complete metabolic pathways into the *C. ljungdahlii* genome. The analysis of additional *C. ljungdahlii* strains possessing *Himar1* transposon mediated genomic integrations revealed a random distribution throughout the genome (unpublished observations). The *C. ljungdahlii* genome is remarkably AT-rich (68.9%) thus offering great variety in terms of the integration site, which allows the exploitation of position effects that could modulate the expression of the integrated transgenes [64]. The xylose-inducible promoter system [33] was useful as a transposase control strategy. Although xylose is metabolized by *C. ljungdahlii* [5] 2% (w/v) of xylose for induction was sufficient and was not metabolized to completion during cultivation (unpublished observations). Recently, an alternative inducible expression system for *C. ljungdahlii* was introduced, based on the *lac* promoter employing lactose, which does not serve as carbon source in *C. ljungdahlii* [65].

Gene transfer to *Clostridium* spp. for the purpose of transgene integration has also been achieved using targeted methods, including the ClosTron system based on group II introns [21, 66], allele coupled exchange [67] and the use of counter-selectable markers during screening [68]. The targeting of specific genomic sites allows these methods to be used for gene knockout and knock-in procedures. Although the ClosTron system is a valuable tool to generate deletion mutants, its efficiency declines when introducing heterologous sequences of more than 1 kb [69]. An elaborate but laborious multistep process system has been used to integrate 40 kb of heterologous DNA into the *C. acetobutylicum* genome [70].

### Expression of the acetone cluster genes and product formation

Finally, having confirmed the integration of an intact cassette in strain Ace#22-24, we used targeted proteomics to confirm the presence of the corresponding heterologous enzymes. Accordingly, in addition to the internal control peptides, we also detected the proteotypic peptides for ThlA, CtfA, CtfB, and Adc, with Adc producing the weakest ion counts (Additional file 4: Figure S4).

**Fig. 4:**
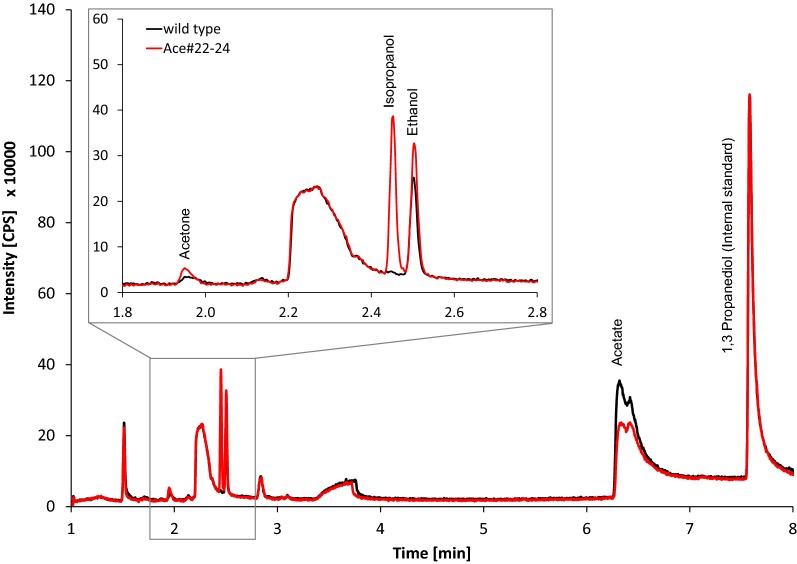
Chromatogram of fermentative products of *C. ljungdahlii* wild type and integration strain Ace#22-24. *C. ljungdahlii* wild type (black) and integration strain Ace#22-24 containing the acetone biosynthesis cluster (red) were grown in modified medium on syngas (33% CO, 33% CO_2_, 33% H_2_) for 2 weeks. The supernatant was analyzed by GC–MS

To verify the activity of the transgene cluster, we measured the amount of acetone and alcohol produced by strain Ace#22-24. Therefore, a serum bottle containing modified ATCC medium was inoculated with the genomic integrant of *C. ljungdahlii* and gassed for 15 s with artificial syngas (comprising an equimolar mixture of CO, CO_2_, and H_2_). After incubation for 2 weeks in a sealed bottle in the syngas atmosphere, the acetone concentration was 0.6 mM, the ethanol concentration was 9 mM, and we also detected 2.4 mM isopropanol (Fig. 4).

In a previous report, the expression of heterologous acetone biosynthesis genes resulted in the production of 13 mM acetone in *C. ljungdahlii* using syngas as the carbon source with an inducible expression system [65]. The difference in final product concentration and profile may reflect differences in the detection methods used or a gene dosage effect, given that our strain contained a single copy of the transgene cassette. The ability of *C. ljungdahlii* to reduce acetone to isopropanol reflects the activity of a secondary alcohol dehydrogenase—such activity has already been detected in *C. ragsdahlii* but not in *C. carboxidivorans* [71]. The corresponding *C. autoethanogenum* and *C. ljungdahlii* enzymes have been characterized more recently [72]. The production of 0.6 mM acetone and 2.4 mM isopropanol by *C. ljungdahlii* strain Ace#22-24 in this study suggests that up to 3 mM acetone could be generated if the alcohol dehydrogenase was inactivated, e.g., by gene knockout. However, isopropanol itself is a valuable substance, since it can be used as a drop-in fuel in order to reduce crystallization temperature of biodiesel [73].

A steadily growing toolbox of strategies becomes available to increase product yields of the desired products in the genus Clostridium. A recent study in *A. woodii* showed the impact of different promoters and different origins of replication (resulting in different plasmid copy numbers) on the heterologous expression of the *C. acetobutylicum* acetone gene cluster, resulting in the accumulation of up to 15.2 mM acetone in sealed serum bottles [74]. Hence, further improvements in the yield of this or other heterologous metabolic pathways might be achieved by selecting strong native, heterologous or even synthetic promoters [75], screening for mutants with different integration sites or multiple copies of the cassette, knocking out competing pathways using CRISPR/Cas9 and related technologies [76], in addition to standard approaches such as medium and process optimization.

## Conclusion

The difficulty of transferring large DNA fragments into the genome of *C. ljungdahlii* represented a major obstacle, which severely limited the metabolic engineering of this industrial relevant organism. In order to obtain products such as acetone/isopropanol or other bio-chemicals and bio-fuels from syngas, a method for transferring metabolic pathways comprising several genes is required. Furthermore, for the purpose of obtaining a stable industrial fermentation strain, genomic integration of heterologous genes is desirable. We were able to transfer plasmids of up to 25.6 kb by conjugation and integrate clusters of up to 11.1 kb into the genome of *C. ljungdahlii*. This method shows the clear advantage over other systems based e.g., on homologous recombination, in that it does not require additional cloning of homology arms or preparation of a target site within the genome of the host strain [68, 77]. In this example, we have demonstrated proof of principle for the construction, conjugal transfer and genomic integration of an acetone biosynthesis pathway in *C. ljungdahlii*. Functionality was confirmed by the formation of acetone and isopropanol from syngas. Our straight-forward strategy for gene delivery and integration provides a basis for the bio-based production of chemicals and fuels using industrial waste streams not only in syngas-fermenting strains but our approach can be easily adapted to other clostridial strains as well.

## Materials and methods

### Bacterial strains and cultivation

A detailed list of bacterial strains used in this study is provided in Additional file 5: Table S1. *E. coli* strains NEB10β and NEB Express (New England Biolabs, Ipswich, MA, USA) were used as conjugation donors. NEB10β was also used for general plasmid cloning procedures. *E. coli* conjugation helper strain Stbl3 was used as part of the GeneArt^®^ Seamless PLUS Cloning and Assembly Kit (Thermo Fisher Scientific, Waltham, MA, USA). This strain contains pRK2013, which provides the *mob* (mobility) and *tra* (transfer) gene functions necessary for efficient conjugation [78]. All *E. coli* strains were cultivated aerobically at 37 °C in LB medium according to Lennox (Carl Roth, Karlsruhe, Germany) on a rotary shaker at 160 rpm. For single-reagent selection, the final antibiotic concentrations were 100 µg/ml ampicillin, 250 µg/ml erythromycin, 34 µg/ml chloramphenicol, and 100 µg/ml kanamycin. For double or triple selection, the antibiotic concentrations were reduced to 50 µg/ml ampicillin, 50 µg/ml erythromycin, and (where applicable) 3.4 µg/ml chloramphenicol. *C. ljungdahlii* DSM-13528 (Deutsche Sammlung von Mikro-organismen und Zellkulturen GmbH, Leibniz, Germany) was cultivated in a Bactron-IV anaerobic workbench (Shellab, Cornelius, OR, USA) under strict anaerobic conditions (5% H_2_, 10% CO_2_, 85% N_2_; Westfalen AG, Münster, Germany). The cells were cultivated without shaking in yeast tryptone fructose (YTF) medium (16 g/l tryptone, 10 g/l yeast extract, 4 g/l sodium chloride (pH 6.0) supplemented with 5 g/l fructose, 0.75 g/l l-cysteine and optionally 2 mg/l resazurin as a redox indicator). Alternatively, modified Protein Expression Technology Center (PETC) 1754 medium (ATCC, Manassas, VA, USA) was used as a defined medium by replacing sodium bicarbonate with 20 mM Bis–Tris. For cultivation on solid plates, we added 1.5% (w/v) BD Difco agar (Becton–Dickinson, Franklin Lakes, NJ, USA). When pouring the liquid cultures into plates, we added 1.5% (w/v) low-melting-point agarose (Carl Roth). Low-level oxygen contamination was detected on agar plates prepared as above (omitting l-cysteine but including resazurin) by incubating them in an anaerobic atmosphere. Where applicable, antibiotics were used at the following concentrations: 4 µg/ml clarithromycin, 4 µg/ml thiamphenicol, and 200 µg/ml d-cycloserine (the latter to counter-select against *E. coli*).

### Vector construction

Shuttle vector pSOS-MCS is based on pSOS95 (GenBank AY187686.1; [79]). A *BamH*I-*Nar*I fragment containing the *ctfA*-*ctfB*-*adc* cassette was replaced with the multiple cloning site from pYUBDuet, prepared with the same enzymes. A cluster containing the acetone biosynthesis pathway was designed, encompassing the genes for acetoacetate decarboxylase (*adc*, CA_P0165, *Clostridium beijerinckii*), acetyl-CoA acetyltransferase (*thl*A CA_C2873, *C. acetobutylicum*), CoA-transferase subunit A (*ctfA*, CA_P0163, *C. acetobutylicum*), and CoA-transferase subunit B (*ctfB*, CA_P0164, *C. acetobutylicum*). The genes were synthesized by GenScript (Piscataway, NJ, USA) and integrated into pSOS-MCS by Gibson assembly [80] to generate vector pSOS-MCS-acetone. An *oriT* region including the adjacent *traJ* gene was integrated in the same manner to generate vector pSOS-MCS-traJ. The origin of replication *repL* in the vector was replaced with *repH* [40]. The pSOS-MCS-traJ and *repH* sequences were amplified by PCR using primer pairs pSOS-traJ del repL for/rev and repH-pSOS-SLIC for/rev, respectively, and the products were joined by isothermal in vitro recombination [80] to produce the final vector pSOS-traJ-repH.

The pIM vector was assembled from four synthetic fragments (GenScript) and an *oriT* region with the adjacent *traJ* gene were inserted by Gibson assembly resulting in pIM-traJ. The shuttle vector pIM-SLIC was derived from vector pIM-traJ to support sequence and ligation independent cloning (SLIC) and Gibson assembly [80]. Therefore, a *Sac*II fragment of pET41a containing the multiple cloning sites was prepared and inserted into pIM-traJ, which had been linearized with *Sac*II. Plasmid pIM-SLIC was used to introduce the amino acid exchanges for the hyperactive *Himar1* C9 transposase version [58] by Quick Change PCR with primers QC_himar_for1 and QC_himar_rev1. Subsequently, the *repH* origin of replication was introduced by Gibson assembly using fragments amplified by primer pairs pIM-repH SLIC for1/rev2 for repH and pIM-repH SLIC for2/rev1 for the remaining backbone resulting in pIM-SLIC-H1C9-repH.

The pIM-Ace#22 vector was prepared by amplifying the complete acetone gene cluster in pSOS-MCS-acetone using Herculase II DNA polymerase (Agilent Technologies, Santa Clara, CA, USA) and primers Ace_096 and Ace_087, resulting in a 3.3-kb product. Vector pIM-SLIC was amplified as above using primers Gn_026 and Gn_027. The products were combined by isothermal in vitro recombination at 50 °C. The primers used in this study are listed in Additional file 6: Table S2.

### Conjugation (triparental mating)

A pre-culture of *C. ljungdahlii* inoculated from an exponentially growing culture was cultivated overnight in YTF medium and diluted the following morning to an OD_600_ of 0.05. The diluted culture was incubated anaerobically at 37 °C and split successively into several tubes depending on the number of planned conjugations (40 ml each) and was grown until the OD_600_ reached 0.2–0.3.

In the meantime, an inoculation loop full of *E. coli* helper strain Stbl3 with pRK2013 and donor strain NEB Express or NEB 10β containing the plasmid to be conjugated were transferred from overnight agar plates into separate shake flasks containing 100 ml and 50 ml LB medium, respectively, supplemented with the appropriate antibiotics. The cultures were incubated until the OD_600_ reached 0.2–0.5, then, for each conjugation 10 ml of the helper strain and 40 ml of the donor strain were harvested by centrifugation (4000*g*, 10 min, room temperature), washed with fresh LB medium to remove residual antibiotics and centrifuged at 12,000*g* for 1 min at room temperature. The cells were then washed with anaerobic YTF medium to remove residual oxygen, centrifuged and resuspended in 100–200 µl fresh YTF medium.

The *E. coli* donor and helper strains were mixed and incubated at 37 °C anaerobically, and meanwhile the *C. ljungdahlii* cells were recovered by centrifugation (4000*g*, 15 min, room temperature) in tightly closed 50-ml Corning tubes. The supernatant was carefully discarded and the pellet resuspended in the residual medium and pooled. Aliquots were added to the *E. coli* mixed culture to initiate triparental mating. The relative proportions of the three strains were equivalent to OD_600_ values (in total) of ~ 5 for the *E. coli* helper strain, 2–20 for the *E. coli* donor strain and 4.5–12 for the recipient *C. ljungdahlii* strain. The cell suspension was spread onto YTF agar plates without selection and allowed to dry completely at 37 °C for 12–20 h. The cells were resuspended by adding 2–3 ml fresh medium to the agar surface and dislodging the cells with an inoculation loop. The suspension was transferred to Petri dishes and mixed with YTF plus 1.5% (w/v) low-melting-point agarose at ~ 37 °C, which was allowed to solidify at room temperature for 1 h before transfer to 37 °C (Bactron-IV). The first colonies became visible after 3–7 days, depending on the origin of replication and concentration of antibiotics.

### Genomic integration

Single colonies were inoculated into YTF medium containing 4 µg/ml clarithromycin, 4 µg/ml thiamphenicol, and 200 µg/ml d-cycloserin. After verification by plasmid rescue and PCR sequencing using primers 16S_fD1 and 16S_rD1 [81], gene cluster integration was induced by transferring the cells to YTF medium as above but lacking thiamphenicol and containing 2% (w/v) xylose. The cells were subcultured at least three times in Hungate tubes. Single colonies were obtained by streaking on YTF agar plates or by spreading 2-µl aliquots into YTF agar plates without clarithromycin, thiamphenicol, and xylose. Plasmid loss was confirmed by replica plating onto agar plates without antibiotics, or supplemented with clarithromycin alone, or supplemented with clarithromycin and thiamphenicol.

### Inverse PCR

To confirm integration, genomic DNA was isolated using the Genomic DNA from Tissue Kit (Macherey–Nagel, Düren, Germany) according to the manufacturer’s recommendations for difficult-to-lyse bacteria, with one exception: after treatment with lysozyme (20 mg/ml) in lysis buffer (20 mM Tris/HCl, 2 mM EDTA, 1% Triton X-100, pH 8.0) the cells were resuspended in fresh lysis buffer (without lysozyme) before adding proteinase K. From the isolated genomic DNA 500 ng was digested with different restriction enzymes (1 µl of an appropriate restriction enzyme, e.g., *Ase*I, *Bsr*GI, *Hin*dIII, or *Pst*I (New England Biolabs) in 30 µl buffer for 6 h) and 5 µl of the heat-inactivated reaction mixture was used for ligation in a reaction volume of 20 µl containing one unit of T4 DNA ligase (Thermo Fisher Scientific) at 15 °C overnight. The products were amplified from 1 µl of a 1:20 dilution using Herculase II DNA polymerase and primers iPCR_mlsR_for01 and iPCR_mlsR_rev01 for PCR (initial denaturation at 95 °C for 5 min, 40 cycles of denaturation at 95 °C for 30 s, annealing at 53 °C for 30 s, and elongation at 72 °C for 3 min 30 s, then a final elongation step at 72 °C for 5 min before holding at 4 °C). The PCR products were verified by in-house sequencing using an ABI 3730 DNA Analyser (Applied Biosystems, Thermo Fisher Scientific).

### Methylation analysis and whole-genome sequencing

The cytosine methylation status of the *C. ljungdahlii* genome was characterized by bisulfite sequencing [82] using a 10-µg aliquot of genomic DNA obtained from stationary cell cultures. Bisulfite conversion and sequencing was carried out by Zymo Research (Irvine, CA, USA). Methylated adenines were detected by PacBio single-molecule, real-time DNA sequencing [83]. Genomic DNA was prepared from wild-type cultures in the logarithmic growth phase (OD_600_ = 0.6) and sequenced by Microsynth (Balgach, Switzerland). A SMRTbell™ library was prepared according to the 10-kb template protocol using the DNA Template Kit 2.0, and was sequenced using P4-C2 chemistry on an RSII instrument (all from Pacific Biosciences, Menlo Park, CA, USA). The whole-genome sequencing of integrant Ace#22-24 was carried out by Seq-IT GmbH & Co KG (Kaiserslautern, Germany) using 2 µg of genomic DNA. The library was prepared using the Nextera XT DNA Sample Preparation Kit (Illumina, Inc., San Diego, CA, USA) and was sequenced using a MiSeq Benchtop Sequencer (Illumina) generating paired-end reads of 2 × 250 bp with a coverage of ~ 20–100×.

### Protein extraction and LC–MS/MS analysis

Protein samples were prepared as previously described [84] with minor modifications. Briefly, 35 ml of *C. ljungdahlii* culture grown in modified ATCC 1754 medium with 0.5% (w/v) fructose was harvested by centrifugation (4000*g*, 15 min, 4 °C) and the pellet was resuspended in 1 ml freshly prepared 50 mM ammonium bicarbonate (pH 7.8). The cells were disrupted by milling with 500 mg 0.1-mm zirconia/silica beads (Carl Roth). After centrifugation (13,000*g*, 2 min, 4 °C) the soluble protein fraction was transferred into a fresh Eppendorf tube. The protein concentration was determined using the Bradford assay, and an aliquot containing 200 µg total protein was then processed, digested with trypsin and desalted as previously described [84]. The digested samples were analyzed by LC–MS/MS using a Shimadzu UFLC (Shimadzu Corp., Kyoto, Japan) fitted with a Supelco Ascentis^®^ Express Peptide ES-C18 reversed-phase column (50 × 2.1 mm, 2.7 µm particle size; Sigma-Aldrich, Munich, Germany). Fractionated samples were injected into a QTRAP6500 triple-quadrupole mass spectrometer (AB Sciex, Framingham, MA, USA) managed with Analyst software v1.6.2. The following proteotypic peptides were used to confirm the presence of the acetone cluster: APYLANNAR (ThlA), TGLGTLIEK (CtfA), NTTIDEIR (CtfB), and EYLNIIYR (Adc). The following proteotypic peptides were used as *C. ljungdahlii* internal controls: DFEPVLER (CODH/ACS, CO dehydrogenase/acetyl-CoA synthase complex beta subunit, CLJU_c37550), AIEAGLK (MeTr, methyltetrahydrofolate:corrinoid/iron-sulfur protein, CLJU_c37560), and IVVGYTR (F-THFL, formate-tetrahydrofolate ligase, CLJU_c37650). Peptide FDGTVEVK (glyceraldehyde 3-phosphate dehydrogenase) was used to exclude potential *E. coli* contamination.

### GC–MS analysis

The concentrations of acetone, isopropanol, ethanol, and acetate were determined by GC–MS analysis using a Shimadzu GCMS-QP2010S instrument. Supernatants from bacterial cultures were diluted 1:10 in 100% methanol spiked with 5.5 mM 1,3-propanediol as an internal standard, and were fractionated on an InertCap FFAP capillary column (0.25 mm × 30 m, 0.25 µm film thickness; GL Sciences, Torrance, CA, USA). A 1-µl sample was evaporated at 200 °C, and the following temperature profile was then applied: initial 3 min hold at 50 °C, temperature gradient (35 °C/min) to 220 °C, and final hold at 220 °C for 2 min. The eluate was ionized at 1 kV and the total ion count was monitored. Absolute concentrations of analytes were calculated using corresponding calibration curves.

## Additional files

**Additional file 1: Figure S1.** Restriction digest analysis of re-isolated plasmid pIM-Ace#22. To verify the complete transfer of pIM-Ace#22 into *C. ljungdahlii* by conjugation the plasmid was isolated and retransformed into *E. coli*. An agarose gel from pIM Ace#22 and the reisolated plasmid is shown after restriction digest with *Eco*RV, *Sca*I and *Xba*I.

**Additional file 2: Figure S2.** Inverse PCR on genomic integrant strain Ace#22-24. To identify the site of integration of the acetone biosynthesis pathway including the resistance cassette into the genome of strain Ace#22-24 an inverse PCR was performed after digestion with the indicated restriction endonucleases and re-ligation. The PCR products were separated and visualized in a 1% agarose gel stained with redsafe.

**Additional file 3: Figure S3.** Analytical PCR for verification of the genomic integrant strain Ace#22-24. Several PCRs were performed on *C. ljungdahlii* wild type strain (wt), integration strain Ace#22-14 (gInt) and controls (K−/K+). Primer pairs used were binding close to the site of integration, on the *catP* resistance gene encoded on the plasmid backbone and the *ermC* resistance gene encoded on the integration cassette between the two ITR sites.

**Additional file 4: Figure S4.** Chromatograms of proteotypic peptides of selected proteins of the Wood–Ljungdahl pathway and the acetone biosynthesis pathway. Protein raw extract was prepared from *C. ljungdahlii* wild type and strain Ace#22-24 with integrated acetone biosynthesis cluster and was subjected to tryptic digest for LC–MS/MS analysis. Targeted proteomics was performed analyzing proteotypic peptides of the Wood–Ljungdahl pathway (MeTr, F-THFL and CODH/ACS) as internal controls and the heterologously expressed acetone biosynthesis pathway (ThlA, CtfA, CtfB and Adc) with at least two transitions (y) per peptide.

**Additional file 5: Table S1.** Organisms used in this study.

**Additional file 6: Table S2.** Oligonucleotides used in this study.
