# Supplementary material for: Development of a metabolic pathway transfer and genomic integration system for the syngas-fermenting bacterium Clostridium ljungdahlii
Source: Biotechnol Biofuels. 2019 May 8;12:112. doi: 10.1186/s13068-019-1448-1 (PMC6507227; doi:10.1186/s13068-019-1448-1)
Supplement: Supplementary file 1 — Additional file 1: Figure S1. Restriction digest analysis of re-isolated plasmid pIM-Ace#22. To verify the complete transfer of pIM-Ace#22 into C. ljungdahlii by conjugation the plasmid was isolated and retransformed into E. coli. An agarose gel from pIM Ace#22 and the reisolated plasmid is shown after restriction digest with EcoRV, ScaI and XbaI. [file 13068_2019_1448_MOESM1_ESM.pptx]

## Slide 1
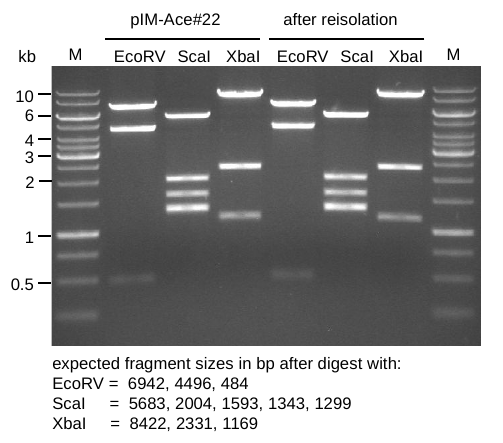

pIM-Ace#22
after reisolation
M
M
kb
EcoRV
ScaI
XbaI
EcoRV
ScaI
XbaI
10
6
4
3
2
1
0.5
expected fragment sizes in bp after digest with:
EcoRV = 6942, 4496, 484
ScaI = 5683, 2004, 1593, 1343, 1299
XbaI = 8422, 2331, 1169
