# Supplementary figures and images for: Development of a metabolic pathway transfer and genomic integration system for the syngas-fermenting bacterium Clostridium ljungdahlii
Source: Biotechnol Biofuels. 2019 May 8;12:112. doi: 10.1186/s13068-019-1448-1 (PMC6507227; doi:10.1186/s13068-019-1448-1)

## Slide 1
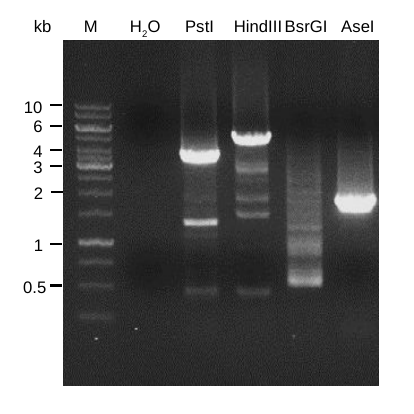

kb
M
H2O
PstI
HindIII
BsrGI
AseI
10
6
4
3
2
1
0.5

Supplement: Supplementary file 2 — Additional file 2: Figure S2. Inverse PCR on genomic integrant strain Ace#22-24. To identify the site of integration of the acetone biosynthesis pathway including the resistance cassette into the genome of strain Ace#22-24 an inverse PCR was performed after digestion with the indicated restriction endonucleases and re-ligation. The PCR products were separated and visualized in a 1% agarose gel stained with redsafe. [file 13068_2019_1448_MOESM2_ESM.pptx]

## Slide 1
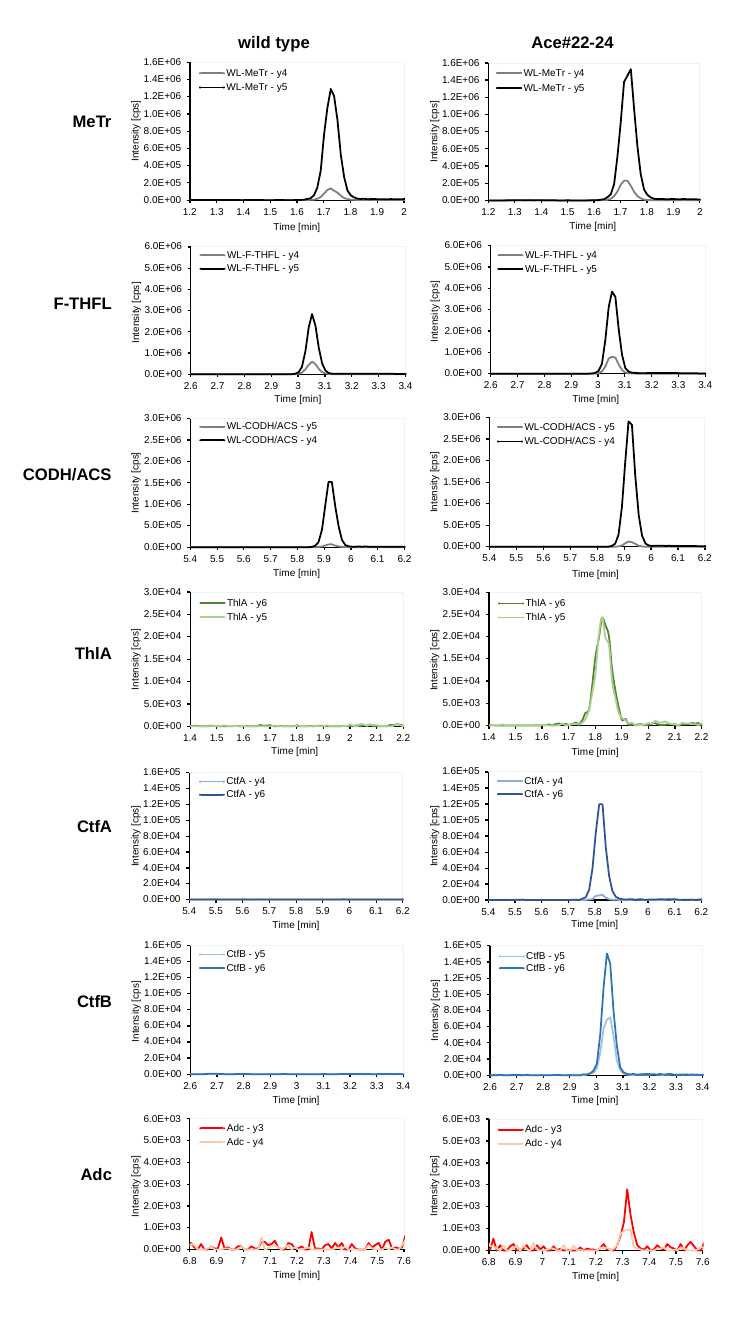

wild type
Ace#22-24
MeTr
F-THFL
CODH/ACS
ThlA
CtfA
CtfB
Adc

Supplement: Supplementary file 4 — Additional file 4: Figure S4. Chromatograms of proteotypic peptides of selected proteins of the Wood–Ljungdahl pathway and the acetone biosynthesis pathway. Protein raw extract was prepared from C. ljungdahlii wild type and strain Ace#22-24 with integrated acetone biosynthesis cluster and was subjected to tryptic digest for LC–MS/MS analysis. Targeted proteomics was performed analyzing proteotypic peptides of the Wood–Ljungdahl pathway (MeTr, F-THFL and CODH/ACS) as internal controls and the heterologously expressed acetone biosynthesis pathway (ThlA, CtfA, CtfB and Adc) with at least two transitions (y) per peptide. [file 13068_2019_1448_MOESM4_ESM.pptx]
