# Supplementary material for: Development of a metabolic pathway transfer and genomic integration system for the syngas-fermenting bacterium Clostridium ljungdahlii
Source: Biotechnol Biofuels. 2019 May 8;12:112. doi: 10.1186/s13068-019-1448-1 (PMC6507227; doi:10.1186/s13068-019-1448-1)
Supplement: Supplementary file 3 — Additional file 3: Figure S3. Analytical PCR for verification of the genomic integrant strain Ace#22-24. Several PCRs were performed on C. ljungdahlii wild type strain (wt), integration strain Ace#22-14 (gInt) and controls (K−/K+). Primer pairs used were binding close to the site of integration, on the catP resistance gene encoded on the plasmid backbone and the ermC resistance gene encoded on the integration cassette between the two ITR sites. [file 13068_2019_1448_MOESM3_ESM.pptx]

## Slide 1
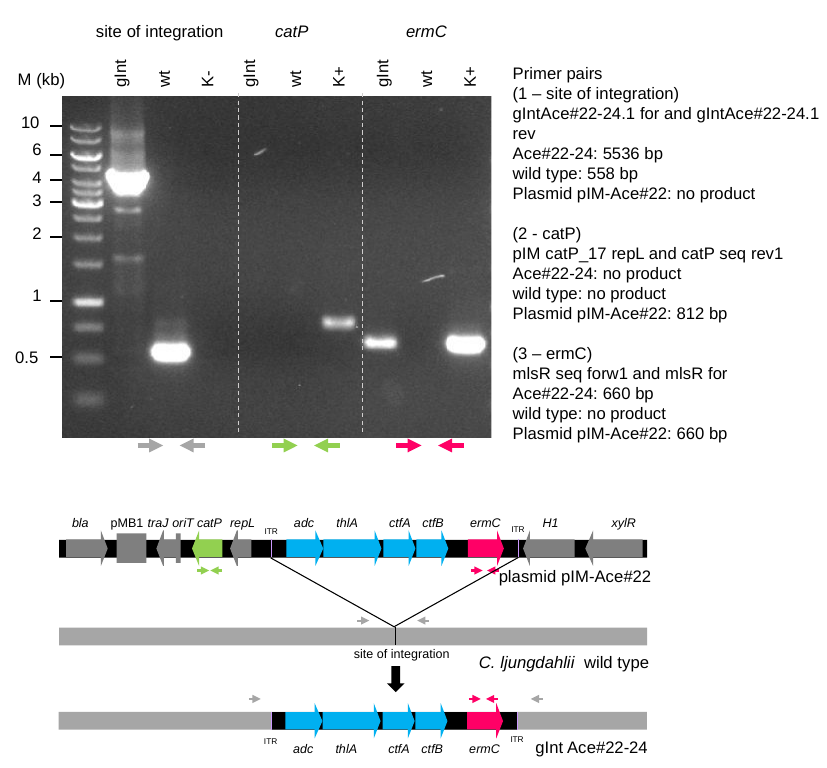

site of integration
catP
ermC
gInt
wt
K-
gInt
wt
K+
gInt
wt
K+
Primer pairs
(1 – site of integration)
gIntAce#22-24.1 for and gIntAce#22-24.1 rev
Ace#22-24: 5536 bp
wild type: 558 bp
Plasmid pIM-Ace#22: no product
(2 - catP)
pIM catP_17 repL and catP seq rev1
Ace#22-24: no product
wild type: no product
Plasmid pIM-Ace#22: 812 bp
(3 – ermC)
mlsR seq forw1 and mlsR for
Ace#22-24: 660 bp
wild type: no product
Plasmid pIM-Ace#22: 660 bp
M (kb)
10
6
4
3
2
1
0.5
bla
pMB1
traJ
oriT
catP
repL
adc
thlA
ctfA
ctfB
ermC
H1
xylR
ITR
ITR
plasmid pIM-Ace#22
C. ljungdahlii wild type
site of integration
gInt Ace#22-24
ITR
ITR
adc
thlA
ctfA
ctfB
ermC
