# Supplementary material for: Development of a metabolic pathway transfer and genomic integration system for the syngas-fermenting bacterium Clostridium ljungdahlii
Source: Biotechnol Biofuels. 2019 May 8;12:112. doi: 10.1186/s13068-019-1448-1 (PMC6507227; doi:10.1186/s13068-019-1448-1)
Supplement: Supplementary file 5 — Additional file 5: Table S1. Organisms used in this study. [file 13068_2019_1448_MOESM5_ESM.docx]

Table S1. Organisms used in this study

| Name | Genotype | Reference |
| --- | --- | --- |
| *Clostridium ljungdahlii*  DSM 13528 | wild type | Deutsche Sammlung von Mikroorganismen und Zellkulturen (DSMZ) |
| *E. coli* NEB10β | *araD*139 ∆(*ara*-*leu*)7697 *fhuA* *lacX74* *galK* (ϕ80 ∆(*lacZ*)*M15*) *mcrA* *galU recA1* *endA1* *nupG rpsL* (Str^R^) ∆(*mrr-hsdRMS-mcrBC*) | New England Biolabs (C3019) |
| *E. coli* NEB Express | *fhuA2* [*lon*] *ompT* *gal* *sulA11* *R*(*mcr*-*73::miniTn10*--Tet^S^)*2* [*dcm*] *R*(*zgb-210::Tn10*--Tet^S^) *endA1* Δ(*mcrC-mrr*)*114::IS10* | New England Biolabs (C2523) |
| *E. coli* dam-/dcm- | *ara-14 leuB6 fhuA31 lacY1 tsx78 glnV44 galK2 galT22 mcrA dcm-6 hisG4 rfbD1* *R*(*zgb210::Tn10*) Tet^S^ *endA1 rspL136* (Str^R^) *dam13::Tn9* (Cam^R^) *xylA-5 mtl-1 thi-1 mcrB1 hsdR2* | New England Biolabs (C2925) |
| *E. coli* ER2739 (GM4714) | *trp-31 his-1 fhuA2 rpsL104*(Str^R^) Δ(*lacZ*)*r1* *glnV44 xyl-7 mtl-2 metB1* *mcr-62 argG6* Δ(*mcrB-hsd-mrr*)*114::IS10 dam-16::Kan* | Elisabeth Raleigh, New England Biolabs)  [1] |
| *E. coli* Stbl3 with pRK2013 | *F– mcr*B *mrr hsd*S20(r_B_*^–^,* m_B_^–^) *recA13 supE44 ara-14 galK2 lacY1 proA2 rpsL20*(Str^R^) *xyl-5 λ– leu mtl-1*  pRK2013: 48kb, Kan^R^, RK2 replicon | Thermo Fisher Scientific  [2] |
|  |  |  |

1. Palmer BR, Marinus MG: The *dam* and *dcm* strains of *Escherichia coli*--a review. *Gene* 1994, 143:1-12.

2. Figurski DH, Helinski DR: Replication of an origin-containing derivative of plasmid RK2 dependent on a plasmid function provided in trans. *Proc Natl Acad Sci U S A* 1979, 76:1648-52.
