# Supplementary material for: Development of a metabolic pathway transfer and genomic integration system for the syngas-fermenting bacterium Clostridium ljungdahlii
Source: Biotechnol Biofuels. 2019 May 8;12:112. doi: 10.1186/s13068-019-1448-1 (PMC6507227; doi:10.1186/s13068-019-1448-1)
Supplement: Supplementary file 6 — Additional file 6: Table S2. Oligonucleotides used in this study. [file 13068_2019_1448_MOESM6_ESM.docx]

Table S2. Oligonucleotides used in this study

| Name | Sequence 5′→ 3′ | purpose |
| --- | --- | --- |
| 16S_fD1  16S_rD1  iPCR_mlsR_for01  iPCR_mlsR_rev01 | ccgaattcgtcgacaacAGAGTTTGATCCTGGCTCAG  cccgggatccaagcttAAGGAGGTGATCCAGCC  AGCACGAGCTCTGATAAATATGAAC  ACATGCAGGAATTGACGATTTAAAC | verification of *C. lju*  verification of *C. lju*  inverse PCR  inverse PCR |
| pSOS-traJ del repL for | GCAAAAGACATAATCGATTCAC | cloning |
| pSOS-traJ del repL rev | GCGATTAAGCTCAAGCTTG | cloning |
| repH-pSOS-SLIC for | CGACGGCCAGTGCCAAGCTTGAGCTTAATCGCACTGGCCGGCCGCTTATAATCCATAACAATC | cloning |
| repH-pSOS-SLIC rev | GTGTACCTATTTTTTGTGAATCGATTATGTCTTTTGCATGGCGCGCCGCCATTATTTTTTTG | cloning |
| Ace_096 (Adc-thlaRBS-pET-SLIC for ) | GTACCGGTGGTGGCTCCGGTGATGACGACGACAAGAGGAGGTTAGTTAGAATGTTAGAAAGTGAAGTATCTAAACAAATTAC | cloning |
| Ace 087 (CtfB-pET-SLIC rev) | GGCTTTGTTTAGCAGCCTAGGTATTAATCAATTAGCTAAACAGCCATGGGTCTAAG | cloning |
| Gn_026 (pET ampl for) | CTTGTCGTCGTCATCACCGGAGCCACCACCGGTAC | cloning |
| Gn_027 (pET ampl rev) | CTAATTGATTAATACCTAGGCTGCTAAACAAAGCC | cloning |
| gIntAce#22-24.1 for | TTCGTAACATCAAGCCTTTTATTTTGTG | check PCR |
| gIntAce#22-24.1 rev | TATTCCTGCATATAAGTTGAGTTTTAAG | check PCR |
| pIM catP_17 repL | GTAGTTATTGGGAGGTCAATCTATG | check PCR |
| catP seq rev1 | TAGGGTAACAAAAAACACCG | check PCR |
| mlsR seq forw1 | GTTTATGCATCCCTTAACTTAC | check PCR |
| mlsR for | GATAATATCTTTGAAATCGGCTCAGG | check PCR |
| pIM-repH SLIC for1 | CTGGCGCGCCGCCATTATTTTTTTGAACAATTGACAATTCATTTCTTATTTTTTATTAAGTGATAG | cloning |
| pIM-repH SLIC for2 | GATGATTGTTATGGATTATAAGCGGCCGGCCAGTGGGCAAGTTGAAAAATTCACAAAAATGTGG | cloning |
| pIM-repH SLIC rev1 | GAAATGAATTGTCAATTGTTCAAAAAAATAATGGCGGCGCGCCAGAGCCTACGAGTTCCGAACTAG | cloning |
| pIM-repH SLIC rev2 | TTGCCCACTGGCCGGCCGCTTATAATCCATAACAATCATCCTTTCTGTGACACTGTCAGACAC | cloning |
| QC_himar_for1 | ACAA**AG**ACGTGTTGATGATTCT**A**AGCGGTGTTTGCAGCTGTTAACTC | cloning |
| QC_himar_rev1 | CACCGCT**T**AGAATCATCAACACGT**CT**TTGTTTTTGGTCAAATGTGAG | cloning |
